# A cross-sectional analysis of falsified, counterfeit and substandard medicines in a low-middle income country

**DOI:** 10.1186/s12889-020-08897-x

**Published:** 2020-05-20

**Authors:** Daariimaa Khurelbat, Gereltuya Dorj, Bruce Sunderland, Tsetsegmaa Sanjjav, Enkhtuul Bayarsaikhan, Davaadagva Damdinjav, Gantuya Dorj, Altantuya Jigjidsuren, Oyun Lkhagvasuren, Baasandorj Erdenetsetseg

**Affiliations:** 1 School of Pharmacy, Mongolian National University of Medical Sciences, S. Zorig street, Ulaanbaatar, Sukhbaatar District 14210 Mongolia; 2 School of Pharmacy, Curtin University of Technology, GPO Box U1987, Perth, 6845 Western Australia; 3 School of Public Health, Mongolian National University of Medical Sciences, S. Zorig street, Ulaanbaatar, Sukhbaatar District 14210 Mongolia; 4 Asian Development Bank, Mongolia Resident Mission, Ulaanbaatar, Sukhbaatar District 14210 Mongolia

**Keywords:** Essential medicine, Falsified and substandard medicines, Quality, Mongolia

## Abstract

**Background:**

High prevalence of falsified, counterfeit and substandard medicines pose a threat to public health and treatment failure. This study aimed to investigate the quality of selected essential medicines available in Mongolia.

**Methods:**

A cross-sectional study collected essential medicines from pharmacy outlets in Mongolia, during June and July, 2017. These products were then submitted for pharmacopoeial analysis and registration status.

**Results:**

A total of 1770 samples from 118 pharmacy entities were purchased from wholesalers in urban and rural areas. Pharmacopoeial analysis found 179 (10.1%) samples or eleven product groups were unacceptable. The prevalence of substandard locally produced medicines (n = 105, 5.9%) was higher than imported equivalents [(n = 74, 4.17%, p = 0.0001)]. Approximately one-third of all unacceptable tests were related to assay (n = 73, 30.8%) and weight variation. Of 1770 samples, 76 (4.3%) were unregistered and the prevalence of unregistered samples was 3.8% in Ulaanbaatar city and 5.8% in rural areas, respectively.

**Conclusions:**

This study has indicated that falsified and substandard medicines are prevalent in Mongolia. Considerable effort is required by regulatory authorities, private manufacturers, as well as importers to increase the quality of essential medicines in Mongolia.

## Background

The availability of low quality and/or counterfeited pharmaceutical products is one of the major barriers to provide quality essential health care in developing countries [1, 2]. Indeed it is also an issue in some high-income countries [3, 4].

According to the World Health Organization (WHO), substandard medicines also called “out of specification”, are authorized medical products that fail to meet their quality standards or specifications, or both. Unregistered medical products have not undergone an evaluation and obtained approval by the National Regulatory Authority for marketing. Falsified medical products deliberately/fraudulently misrepresent their identity, composition or source [5].

Recently, Ozawa et al. completed a systematic review of 44 extracted prevalence studies, conducted in 25 different countries. The median prevalence of substandard/counterfeit medicines was 28.5% (11–48%) [6]. Comparable findings were also reported by the WHO in 2017 [7] and in other studies [8–10]. Moreover, the WHO has estimated the cost to procure substandard and falsified medicines to be approximately US$30.5 billion worldwide [7]. Substandard and falsified medicines impact the community at patient-level, and if antibiotics pose a risk of antimicrobial resistance, cause ineffective treatment outcomes and unnecessary increased cost and health burdens [11].

The WHO reported surveillance of antibiotic consumption data worldwide and Mongolia was one of the countries with the highest antibiotic consumption (64.4 DDD per 1000 inhabitants per day) [12]. A pilot study of counterfeit medicines in Mongolia was undertaken by the Ministry of Health (MOH) of Mongolia and 3.5% were considered possibly counterfeit [13]. A larger scale study at the nationwide level indicated a higher prevalence of 14.6% (13.2–17.8) in 2012. The mission of the National Medicines Policy of Mongolia (NMPM) is to provide continuously, equitable and adequate supplies of medications for individuals, health facilities and veterinary services which are effective, safe, of good quality and affordable; and to promote the rational use of medicines [14]. Nevertheless, despite the Government’s efforts to achieve successful implementation of the NMPM [14], the situation has deteriorated with respect to availability [15, 16], quality [13, 17, 18] and rational use [19, 20].

The current study aimed to perform a repeat survey to assess and compare the quality of current medicines, including those manufactured in Mongolia.

## Methods

A cross-sectional study followed the WHO Guidelines on the “Conduct of Surveys of the Quality of Medicines” [21]. This methodology was adapted to the Mongolian context, in particular the selection of medicines and pharmacy entities.

### Quality analysis of samples

#### Materials for quality assessment

Quality assessment tests including (i) appearance, (ii) weight variation, (iii) hardness, (iv) friability, (v) disintegration time and (vi) assay were determined according to pharmacopoeial methods according to the origin of the product or specification requirements from the manufacturer. In Mongolia, several pharmacopeias including the Mongolian National Pharmacopoeia (MNP), developed in 2011 and other relevant documents such as British Pharmacopoeia (BP), Pharmacopeia of the People’s Republic of China (CP), United States Pharmacopeia (USP) and European Pharmacopeia (EP) are accepted as quality control standards.

USP, BP and CP reference standards of acetylsalicylic acid, amlodipine, cefotaxime, cetirizine, ciprofloxacin, ibuprofen, metronidazole, omeprazole, and sildenafil citrate were generously donated by the manufacturers. These included Daewon Pharm Co. Ltd. (Seoul, Korea), Kyongbo Pharmaceutical Co., Ltd. (Seoul, Korea), Sigma Aldrich (USA), Alchemy Medicine Pvt. Ltd. (India), Aristo Pharmaceuticals (Bangladesh), Abaris Healthcare Pvt. (India), Aristo Pharmaceuticals (India), Truong Tho Pharma Isc (Vietnam), NCPC (China). All other chemicals were commercially available and of analytical grade.

#### Assessment of quality

Identification of assay impurities was performed by Thin-Layer-Chromatography (TLC) initially [22, 23]. The system employed glass -backed 5 × 10 cm silica gel 60 F254 plates (E. Merck, Darmstadt, Germany), development of appropriate mobile phases in standard TLC containers, detection of black fluorescence-quenched spots on a bright green background under 254 nm UV light and of brown spots in white light after dipping the plate in the appropriate solution contained in a plastic bag.

Any suspicious samples and their active ingredients were identified by HPLC using UV detection (ThermoFisher Scientific Ultimate 3000, US). HPLC analysis was completed by using ODS Hypersil 150 × 4.6 mm 5 mkm C18μm column, UV 254 nm detector, flow rate: 1 ml/min, and varying mobile phases depending on the pharmaceutical product.

Quality analysis for the collected samples was performed with reference to USP 36, BP 2013, 2015, CP 2015, national pharmacopeial monograph (NPM), the MNP 1st edition 2011 and registration documents. (Table 1).

**Table 1:** Selected drugs and corresponding reference standards

Name of the drug, dose, dosage form	Reference document
Acetaminophen 500 mg/tab	MNP-2011, NPM-0152-2014, NPM-0046-2013
Acetylsalicylic acid, 81 mg/tab	BP-2013, NPM-0114-2014
Amlodipine 10 mg/tab	NPM-0026-2013, USP-36, Registration document
Amoxicillin 500 mg/tab	BP-2013, CP-2010, MNP-2011,
Cefotaxime 1.0 g/powder for injection	BP-2013, CP-2010, NPM-42-0088298102, NPM-0052-2013, Registration document, USP-36
Cetirizine 10 mg/tab	BP-2013, CP-2010, NPM-0224-2015, Registration document, USP-36
Ciprofloxacin 500 mg/tab	BP-2013, CP-2010, MNP-2011, USP-36
Citramon-P (Acetylsalicylic acid+acetaminophen+caffeine), 450 mg /tab	MNP-2011, NPM-0152-2014, NPM-0154-2014
Diclofenac sodium 100 mg/tab	BP-2013, CP-2010, MNP-2011, NPM-023-2015, NPM-0270-2016, NPM-0115-2014
Ibuprofen, 400 mg /tab	BP-2013, BP-2015, Registration document
Metronidazole 0.5%/100 ml infusion solution	CP-2010, Registration document, USP-36,
Nystatin 500,000 IU/tab	BP-2013, USP-36, Registration document
Omeprazole 20 mg/capsule	BP-2013, Registration document
Panangin (Aspartic Acid, L- potassium and magnesium), 298 mg/tab	Registration document
Sildenafil citrate 100 mg/tab	NPM-0254-2016, Registration document

Note: *BP* British Pharmacopeia, *CP* Chinese Pharmacopeia, *MNP* Mongolian National Pharmacopeia, *NPM* National Pharmacopeial Monograph, *USP* United States Pharmacopeias

The samples were analyzed between August 2017 and December 2017 in the Medicines Quality Control Laboratory, National Reference Laboratory for Food Safety, Generalized Agency for Specialized Inspection of Mongolia (GASI) of Mongolia. The Medicines Quality Control Laboratory, National Laboratory Reference Laboratory for Food Safety, GASI is accredited by the ANSI-ASQ in the field of testing [24].

All analyses of samples were completed within the expiry date for each pharmaceutical product and were stored according to storage requirements for each product immediately after collection.

### Visual inspection and registration status

Visual inspection and registration verification of all samples was conducted in compliance with the WHO recommendations [25] and national regulations [14, 26, 27], .and samples were checked based upon a modified version of the ‘Checklist for the visual inspection of medicines to identify suspicious drug products’. [28] These included evaluation of outer packaging, layout, print color, information regarding the registration number, batch number, manufacturing date and expiry data. A catalogue containing photographs of samples, packaging and package inserts was prepared. Moreover, information regarding the labelling of packages and containers was assessed against a national database, “Licemed” which is maintained and updated by the Center for Health Development, Ministry of Health, Mongolia [29]. The online database “Licemed” contains information regarding the status of registration of pharmaceutical products, in addition photographic images of genuine products registered in Mongolia. In addition, the license status of marketing authorization holders until 31st of January, 2018 of each sample was confirmed by Licemed [29].

Samples with suspicious packaging and labeling were sent to the representative offices in Mongolia of the manufacturers for confirmation whether if the suspected product was their product and discrepancies between the suspected and genuine product were verified.

### Site selection

**Fig. 1:**
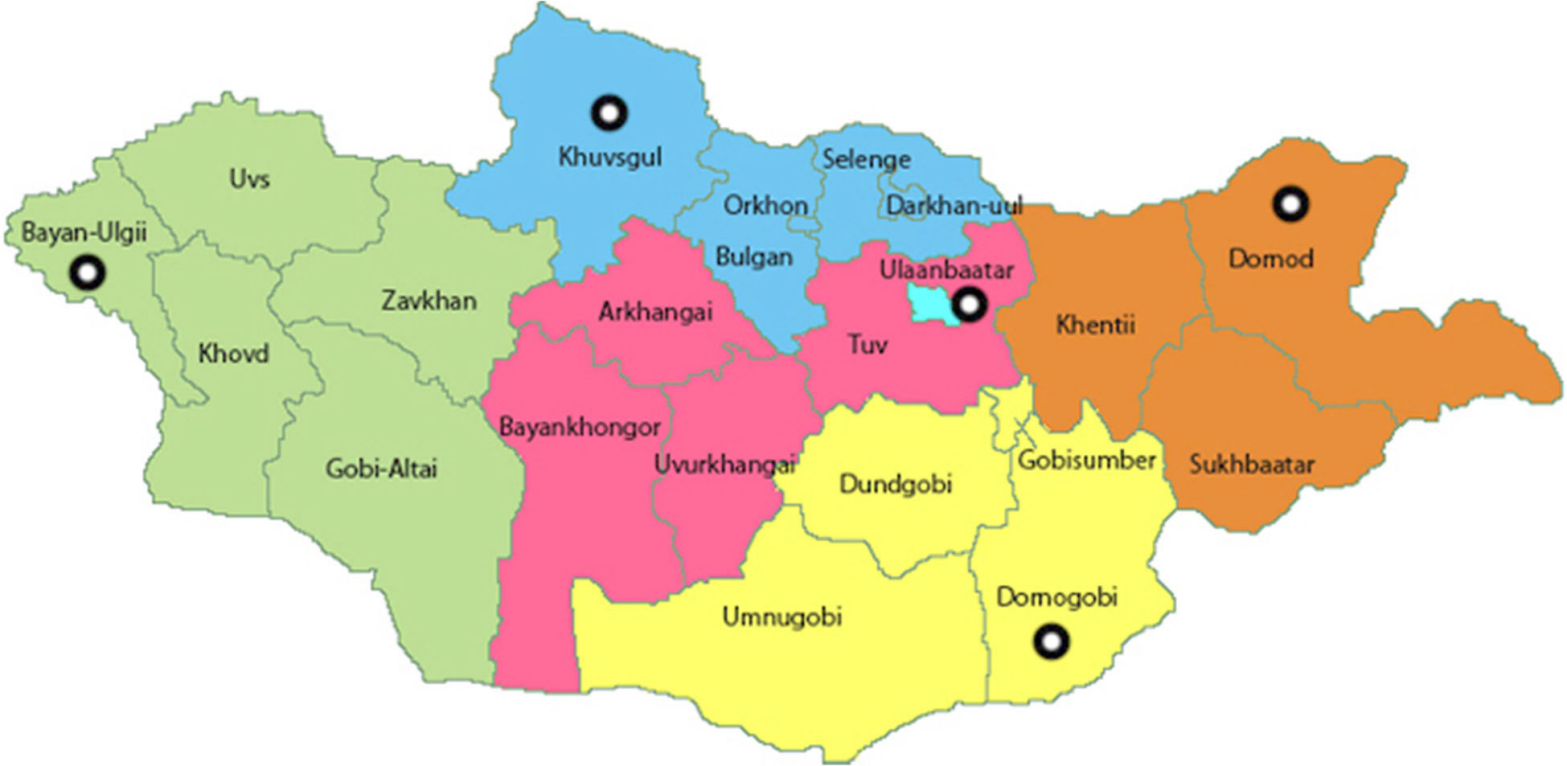
Geographical map of Mongolia and selected regions based upon entry points to Mongolia (downloaded from Mongolia Map by Vemaps.com with authorization)

The study collection sites were divided into two areas: Ulaanbaatar city (the sole urban area) and the rural area. The rural area was divided into four geographical regions, namely Western, Central, Khangai, and Eastern region. From each region, one province was chosen accounting for the risk of transportation of unregistered, falsified and substandard medicines through borders and entry ports between Russia and China, including Bayan-ulgii, Dornogovi, Khuvsgul and Dornod provinces [30]. (Fig. 1).

As for the urban area, four districts, including Songinokhairkhan, Bayanzurkh, Sukhbaatar, Chingeltei, were conveniently selected in Ulaanbaatar and the selection was based on population size and health statistics [31].

### Selection of pharmacy entities

A random method was employed to select the pharmacy entities in these geographical regions. The number of each type of pharmaceutical operations to be included in each region was weighted by the proportion of types of pharmacy entities in that geographic region. Pharmacy entities were selected using a random sample calculator. If the sample could not be obtained from the selected pharmacy entities, another pharmacy entity was randomly selected and substituted in order to make up the required numbers. The list of licensed pharmacy entities was obtained from the General Health Department of Ulaanbaatar city and GASI.

### Selection of medicines

Medicines to be sampled were selected based on discussions with local experts. Specific criteria included that the medicines were included in the Essential Medicine’s List of Mongolia (EML) [32], had been found substandard in previous studies [18], commonly prescribed and dispensed medicines with reimbursement from the health insurance office [33]. Their availability at all types of pharmacy entities, of high therapeutic importance and cost was considered. The medicines selected for sampling were: acetaminophen 500 mg tablets; acetylsalicylic acid 81 mg tablets; amlodipine 10 mg tablets; amoxicillin 500 mg tablets; cefotaxime 1 g vials; cetirizine 10 mg tablets; ciprofloxacin 500 mg tablets; citramon-P (acetylsalicylic acid+acetaminophen+caffeine) 450 mg tablets; diclofenac sodium 100 mg tablets; ibuprofen 400 mg tablets; metronidazole 0.5%/100 ml/infusion; nystatin 5.000.000 IU tablets; omeprazole 20 mg tablets; panangin (aspartic acid, L- potassium and magnesium) 298 mg tablets and sildenafil citrate 100 mg tablets.

### Sampling

Data relating to the collected samples was recorded in pre-developed data collection forms [21, 28] and it included information of the contents of the medicine packages and the pharmacy entity. Sample collection from urban and rural areas was conducted during June and July of 2017 by two teams. In accordance with the WHO guidelines [21], each team consisted of a principal investigator, a locally recruited sampling researcher and an assistant. All members were trained in relation to purchasing the sample medicines according to the sampling procedure. Purchasing medicines and completing sampling forms for the individual medicines was performed by the sampling personnel.

Required numbers of samples of selected medicines were purchased from each pharmacy entity and if the sample was not available in stock, another pharmacy entity was recruited for the study.

All pharmacy entities were coded with a unique number and labeled with the same international non-proprietary name, brand name, strength, size, batch/lot number, and manufacturing and expiry dates for each sample. All containers and packages of medicines were also collected and preserved for confirmation purposes. Samples were maintained as per storage requirements, at 20–25 °C (or as stated on the product) until analysis.

### Sample size calculation

Based on a previous study of falsified/substandard drugs in Mongolia [18], the targeted sample size was to detect at least a 10% prevalence (alpha of 0.05 and beta of 0.9) accuracy. This calculation indicated that 20–30 samples of each drug (300–450 for all drug types combined) from each pharmacy entity were required for this study.

### Data analysis

All data were entered into Microsoft Excel and IBM SPSS Statistics for Windows, version 20 (IBM Corp., Armonk, N.Y., USA). Categorical data were compared by means of Chi-square tests or Fisher’s exact tests. *P* values < 0.05 were considered as statistically significant.

## Results

A total of 1770 samples were purchased from 118 pharmacy entities a majority of which were obtained from retail pharmacies in urban areas (n = 1110, 62.7%). When the samples were not available or there were insufficient samples, the study team randomly selected the next pharmacy entity to make up the samples. From each entity, the same brands, but different batches of pharmaceutical products were purchased and analysed.

Of note, Revolving Drug Funds (RDF) operate only in rural areas and 135 (7.6%) were acquired from RDFs. Similarly, to other areas, the same brands but different batches of products were collected from RDFs.

More samples were purchased from wholesalers in urban areas when compared to their counterparts in rural areas [(n = 210, 11.9% vs n = 60, 3.4%, *p* = 0.001)]. Data regarding individual samples purchased from pharmacy entities in urban and rural areas are presented in Table 2. Samples of one brand could include different batch numbers, due to out-of-stock or unavailability.

**Table 2:** Summary data of collected samples, their sources, strengths and dosage forms

Medicine’s name, dosage, dose form	Sampling area		Pharmacy entity			Sample source	
	Urban	Rural	Wholesaler	Community pharmacy	RDF	Local	Import
Acetaminophen, 500 mg/tab	88	30	43	66	9	118	0
Acetylsalicylic acid, 81 mg/tab	88	30	43	66	9	108	10
Amlodipine, 10 mg/tab	88	30	43	66	9	49	69
Amoxicillin, 500 mg/tab	88	30	43	66	9	45	73
Cefotaxime, 1 g/vial	88	30	43	66	9	14	104
Cetirizine, 10 mg/tab	88	30	43	66	9	9	109
Ciprofloxacin, 500 mg/tab	88	30	43	66	9	14	104
Citramon-P (Acetylsalicylic acid+acetaminophen+caffeine), 450 mg /tab	88	30	43	66	9	102	16
Diclofenac sodium, 100 mg/tab	88	30	43	66	9	73	45
Ibuprofen, 400 mg/tab	88	30	43	66	9	3	115
Metronidazole, 0.5%/100 ml/infusion	88	30	43	66	9	14	104
Nystatin, 5,000,000 IU/tab	88	30	43	66	9	0	118
Omeprazole, 20 mg/tab	88	30	43	66	9	0	118
Panangin (Aspartic Acid, L- potassium and magnesium), 298 mg/tab	88	30	43	66	9	0	118
Sildenafil citrate, 100 mg/tab	88	30	43	66	9	16	102
Total (N = 1770)	1320	450	645	990	135	565	1205

### Visual inspection and authenticity

#### Outer package

In accordance with the drug registration requirements, an observational analysis of the outer package including the layout, print colour and information regarding the printed batch number, registration number, manufacturing, expiry dates were completed. Tablets and capsules were packaged in individual blisters, whereas powder for injection and infusion solutions were packed in sealed bottles, and bags. Of 179 substandard samples, 32 were the same brand but had different batch numbers (Table 3).

**Table 3:** Assessment of the outer packaging for regulatory compliance

Description	Present n (%)	Absent n (%)
Registration number	1694 (95.7%)	76 (4.3%)
Batch number	1761 (99.2%)	15 (0.9%)
Manufacturing date	1761 (99.5%)	15 (0.9%)
Expiration date	1761 (99.5%)	15 (0.9%)

Officially accepted languages of package inserts are Mongolian, Russian or English. Predominantly, Mongolian and English were found, however a small number of the samples was found to be in other languages (n = 23, 1.3%).

#### Pharmacopoeial quality analysis

There were 1770 samples of 15 different medicines analyzed for their quality. Of these 179 (10.1%) samples failed pharmacopoeial tests. An analysis of substandard medicines indicated that the proportion of substandard locally produced medicines (n = 105, 5.9%) was higher than their imported equivalents [(n = 74, 4.2%), *p* = 0.0001]. The prevalence of substandard samples was approximately three times higher for domestic products than imported products (n = 195, 18.7% vs n = 74, 6.1%).

Approximately one-third of all sub-standard pharmacopoeial tests were related to assay (n = 73, 30.8%) and weight variation (Table 4). These results were mostly due to the sub-standard assay content in citramon-P (acetylsalicylic acid+acetaminophen+caffeine), 240 mg + 180 + 30/450 mg tablets) and diclofenac 100 mg tablets with non-compliant weight variation. Samples of omeprazole 20 mg and amoxicillin 500 mg failed due to dissolution test results.

**Table 4:** Number of samples that failed each pharmacopoeial test

Medicine’s name / Test parameter	Appearance	Weight variation	Dissolution	Friability test	Disintegration	Assay of active ingredient	Total tests failed	Total samples failed
Acetaminophen 500 mg	17	8	p	6	p	4	35	23
Acetylsalicylic acid+paracetamol+caffeine 450 mg/tab	p	p	p	p	25	41	66	41
Amlodipine 10 mg	p	8	p	p	p	p	8	8
Amoxicillin 500 mg	p	4	23	p	p	p	27	27
Ciprofloxacin 500 mg	p	11	p	p	p	6	17	17
Diclofenac Sodium 100 mg/tab/cap	p	22	p	p	p	14	36	22
Ibuprofen 400 mg	3	2	p	p	p	p	5	5
Metronidazole 0.5%/100 ml	p	n/a	n/a	n/a	n/a	3	3	3
Nystatin 500,000 IU	10	p	p	p	p	p	10	10
Omeprazole 20 mg	p	7	7	p	p	p	14	7
Sildenafil citrate 100 mg	p	11	p	p	p	5	16	16
Total							237	179

**Note**: n/a-not applicable, p – passed the required test

For all 1770 samples, all mandatory tests required for drug registration in Mongolia were completed in this study. (Table 4).

### Registration status

Of 1770 samples, 76 (4.3%) were unregistered and the prevalence of unregistered samples was 3.8% in Ulaanbaatar city and 5.8% in rural area, respectively. Sildenafil citrate 100 mg tablets (n = 26, 34.2%) and amoxicillin trihydrate 500 mg tablets (n = 22, 22.4%) were found to be the most frequently unregistered samples. All unregistered samples were imported medicines produced by 12 manufacturers from seven different countries.

**Fig. 2:**
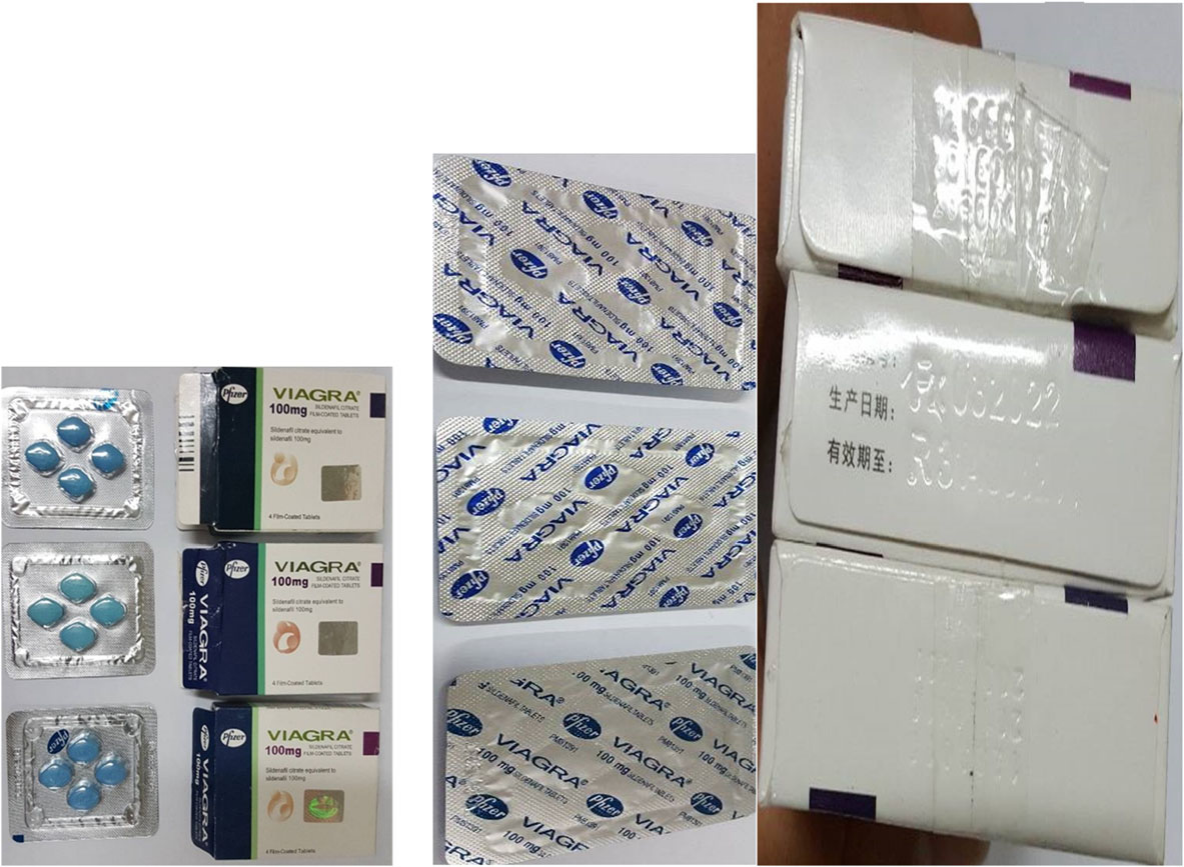
Fake Viagra 100 mg (Sildenafil citrate 100 mg) samples

The proportion of substandard samples were likely to be unregistered (n = 40, 52.6%) when compared to their registered counterparts (n = 139, 8.2%) (*p* < 0.0001). In addition, sildenafil citrate 100 mg was found to be falsified (n = 15, 12.7%) and its manufacturer and country of origin were not identified. Samples did not pass the quantity, content uniformity and information on the outer packaging as well as the tablet appearance. (Fig. 2).

## Discussion

This cross-sectional study investigated the quality of 1770 samples of 15 specific medicines collected from various types of pharmacy entities located in four districts of Ulaanbaatar city and four rural provinces in Mongolia. It was found that 10.1% of the total samples were substandard. In a comparison with a survey performed in 2012 [18], the proportion of substandard medicines had slightly decreased from 14.6 to 10.1% found in this study. However, the prevalence of substandard products was three times higher for locally manufactured products (18.6%) than for imported counterparts (6.1%). Local manufacturing of medicines is promoted in the NMPM and it is mainly to improve the access to essential medicines in Mongolia. However, implementation of good manufacturing practices (GMP) requires significant expenditure for pharmaceutical companies. Large companies operate on a scale that allows them to recover the costs of running high-quality factories, but this is not the case for smaller manufacturers in developing countries [34]. In India, a number of small manufacturers struggle to implement quality-assurance and quality-control procedures even with a large population. Similarly, a majority of locally produced cotrimoxazole were reported to be substandard when evaluated in Indonesia [35].

A systematic review on the prevalence of substandard/falsified medicines from 2007 to 2016 by means of electronic databases reported that it was 10.5% in low-middle income countries. The results in the WHO report are based on studies with the objective to quantify quality problems regarding specific drug classes, including antimalarials and antibiotics [36].

In this study, most of all failed tests were due to assay, content uniformity, dissolution and disintegration. These failures were related to citramon-P (acetylsalicylic acid+acetaminophen+caffeine), 240 mg + 180 + 30/450 mg tablets), omeprazole 20 mg, ciprofloxacin 500 mg and diclofenac sodium, 100 mg tablets owing to poor weight uniformity. Samples of amoxicillin 500 mg and omeprazole 20 mg failed due to dissolution test results in this study.

Apart from being substandard or falsified at the manufacturing stage, the quality of pharmaceutical products is affected by inappropriate storage and transportation conditions during the supply chain [37].

The Mongolian climate is known to be very harsh, with temperatures below − 30 °C in winter. Some rural areas are hot (> 30 °C) in summer. Medicines can deteriorate during storage and transportation in extreme weather conditions and degraded medicines might be distributed in the market [38, 39]. All standard protocols on transportation and storage requirements were complied with during the sample collection and quality assurance was maintained throughout study, including data analysis. However, the samples were collected from different pharmacy entities, meaning that there might be potential risks during distribution or transportation until pharmaceutical products are delivered to each pharmacy entity. Hence, more information to identify the impact of climate during supply and storage chain on drug quality needs to be further investigated.

Good pharmacy practice is promoted by the MOH and enforced by the GASI. However, due to financial constraints and limited human resources, strict quality control measures are yet to be implemented [40, 41].

In this study, one fake sample of sildenafil citrate 100 mg was found. The quantitative content of active ingredient was 45.3–65.4% well below the pharmacopoeial limit (95–105%). Problems with phosphodiesterase type 5 inhibitors (PDE5) have been reported elsewhere, for example an Italian survey on the PDE5 medicines was completed between 2005 and 2011 [42]. It found that 24.0% of the analyzed samples were counterfeit and 54.0% were illegal medicines. In 12.0% of the cases an intermediate classification (illegal/counterfeit) was assigned, whereas only 7.0% of the samples were original [42].

The study showed a decreased prevalence of unregistered medicines, when compared with the previous findings [18]. This could be due to the MOH’s efforts to strengthen the drug registration as well as development of an online database “Licemed” to check and verify drug information. However, discrepancies in outer packaging and information on package inserts can potentially contribute to the inappropriate use of medicines [43].

Research shows that multi-faceted interventions including a mix of regulations, training of inspectors, public-private collaborations and legal enforcement actions are useful in combating substandard and falsified medicines [44]. At the time of writing the manuscript, the regulation of drug registration has been revised and approved by the Mongolian Government [45]. It is envisaged that with revised legal requirements, implementation and enforcement of quality assurance will be improved in Mongolia.

### Limitations

The study aimed to provide a representative sampling in location and size, but only 6.0% of all pharmacy entities, including retail pharmacies, wholesalers and RDFs were included. However, high risk sites and the population size were considered for site selection. A random selection method was employed in selecting the pharmacy entities.

Secondly, samples with suspicious packaging and labeling were sent to the representative offices of the manufacturers for confirmation whether if the suspected product was their product and discrepancies between the suspected and genuine product were verified. However, falsified products can be produced in the same facility as the licensed product. This might be a limitation.

## Conclusion

Overall the national law coordination and law enforcement on registering medicines is in place in Mongolia. Nevertheless, echoing previous findings, the current study results suggest that substandard and falsified medicines are still prevalent in Mongolia. Prevalence of low quality medicines indicate that licensing of manufacturing plants and pharmacy outlets is not fully effective.

### Recommendation

Prevalence of falsified medicines is a major public health problem because it would result in avoidable morbidity, mortality and drug resistance. Regulatory authorities in Mongolia need to enhance their commitment to strengthening licensing of local manufacturers, importers and their GMP compliance. Licensing of local manufacturers should be upgraded to international standards and more stringent rules, including routine quality control tests should be performed to ensure the quality assurance.

## Data Availability

The datasets used and/or analyzed during the current study is from the corresponding author on reasonable request.

## Abbreviations

ADB: Asian Development Bank
ANAB: American National Accreditation Board
ANSI-ASQ: American National Standards Institute and American Society for Quality
BP: British Pharmacopoeia
cap: Capsule
CP: Chinese Pharmacopoeia
EML: Essential Medicines List
EP: European Pharmacopoeia
g: Gramm
GASI: Generalized Agency for Specialized Inspection
IU: International Unit
mg: Milligram
ml: Millilitre
MNP: Mongolian National Pharmacopoeia
MNUMS: Mongolian National University of Medical Sciences
MOH: Ministry of Health
NMPM: National Medicines Policy of Mongolia
RDF: Revolving Drug Fund
Tab: Tablet
WHO: World Health Organization
